# Changes in the availability of medical oxygen and its clinical practice in Ethiopia during a national scale-up program: a time series design from thirty-two public hospitals

**DOI:** 10.1186/s12887-021-02844-4

**Published:** 2021-10-14

**Authors:** Habtamu Seyoum Tolla, Yigeremu Abebe Asemere, Alebel Yaregal Desale, Dinkineh Bikila Woyessa, Zinabie Feleke Fekadu, Alemayehu Berhanu Belete, Audrey Battu, Felix Lam

**Affiliations:** 1 Clinton Health Access Initiative, Addis Ababa, Ethiopia; 2 Clinton Health Access Initiative, Boston, USA

**Keywords:** Medical oxygen, Pulse oximetry, In-patient pediatrics

## Abstract

**Background:**

Oxygen therapy is a lifesaving treatment, however, in Ethiopia, oxygen is not readily available in many healthcare facilities. In 2015, the Federal Ministry of Health launched a national roadmap to increase access to oxygen. This study aims to evaluate whether availability of oxygen and its clinical practice in public hospitals of Ethiopia changed during the time the roadmap was being implemented.

**Methods:**

Between December 2015 and December 2019, a multifaceted approach was undertaken to increase access to oxygen in public facilities in Ethiopia. The activities included formation of new policies, development of guidelines, procurement and maintenance of oxygen equipment, and training of healthcare workers. To evaluate whether access and use of oxygen changed during this period, facility-based surveys were conducted between December 2015 to December 2019. Primary data, including medical record reviews, were collected from 32 public hospitals bi-annually. A chi-square test that claimed *P* < 0.05 used to assess the statistical significance differences.

**Results:**

The study was conducted in 32 public hospitals of Ethiopia, where capacity building and technical support interventions implemented. Of these 32 facilities, 15 (46.9%) were general hospitals, 10 (31.2%) were referral hospitals, and 7 (21.9%) were primary hospitals. Functional availability of oxygen has shown a statistically significant increase from 62 to 100% in the pediatric in-patient departments of general and referral hospitals (*p*-value < 0.001). Similarly, functional availability of pulse oximetry has shown a statistically significant increase from 45 to 96%. With regard to clinical practices, the blood oxygen saturation (SpO2) measurement at diagnosis increased from 10.2 to 75%, and SpO2 measurement at admission increased 20.5 to 83%.

**Conclusions:**

Based on the intervention results, we conclude that multifaceted approaches targeting policy, healthcare workers’ capacity, increased device procurement, and device maintenance programs with on-site mentorship, can improve the availability of medical oxygen and pulse oximetry, as well as clinical practice of oxygen therapy in health facilities. Therefore, ensuring device availability along with regular technical support and close follow-up of healthcare workers and facilities are critical, and these interventions should be scaled further.

**Supplementary Information:**

The online version contains supplementary material available at 10.1186/s12887-021-02844-4.

## Background

Oxygen is an essential medical therapy that can save the lives of many patients [1]. Oxygen therapy is used to treat many diseases which present with hypoxemia, a serious condition caused by low levels of oxygen in the blood [2]. Pneumonia is the largest infectious cause of death in children worldwide, claiming the lives of an estimated 800,000 children under the age of five, which is equivalent to around 2200 children every day [3, 4].

Pneumonia affects developing countries disproportionately. More than 95% of pediatric pneumonia deaths occur in developing countries [5]. In Ethiopia, the prevalence of pneumonia is high, and accounts for 18% of national child mortality. A recent study indicated that pediatric pneumonia prevalence in Ethiopia is 33.5% [6].

Hypoxemia is a common and frequent complication of pneumonia and greatly increases the risk of death [5, 7]. Lack of oxygen quickly leads to dysfunction of the organ system, often resulting in the patients’ death. Hence, hypoxemia is a life-threatening condition that demands early detection and treatment [8].

A systematic review estimated that 13.3% of children with pneumonia have hypoxemia [9]. In addition to pneumonia, the UN IGME report indicated that out of 5.9 million annual child deaths, 23% related to neonatal conditions such as birth asphyxia, sepsis, and low birth weight, can lead to hypoxemia [8]. Therefore, the diagnosis of hypoxemia and administration of oxygen are key strategies in reducing pneumonia and neonatal-related mortality among children under the age of five [2].

However, access to pulse oximeters, a medical device used to diagnose hypoxemia, is limited, and oxygen is often unavailable in countries like Ethiopia. A 2015 assessment of more than 100 hospitals in Ethiopia, showed that for in-patient pediatric departments, only 45% of departments had pulse oximetry available and only 63% of departments had oxygen available. A 2017 clinical review update reported that the use and administration of oxygen in clinical practice is often inappropriate without knowledge of its potential risks and benefits [10]. This is further supported by the findings of a 2016 review on prescription and delivery practice, which reported that, although there have been some changes in oxygen prescription practice, there remains significant room for improvement [11].

To address such challenges, the Clinton Health Access Initiative (CHAI) partnered with the Ethiopian Ministry of Health between 2016 and 2019. The program focused on increasing oxygen availability and use at hospital level, through wide-range of interventions targeting policy and implementation level support.

Therefore, the study aims to assess changes in the functional availability of oxygen devices and clinical practices of oxygen therapy in in-patient pediatrics department from public hospitals in Ethiopia during the implementation of interventions.

## Methods

### Oxygen strengthening program

**Fig. 1:**
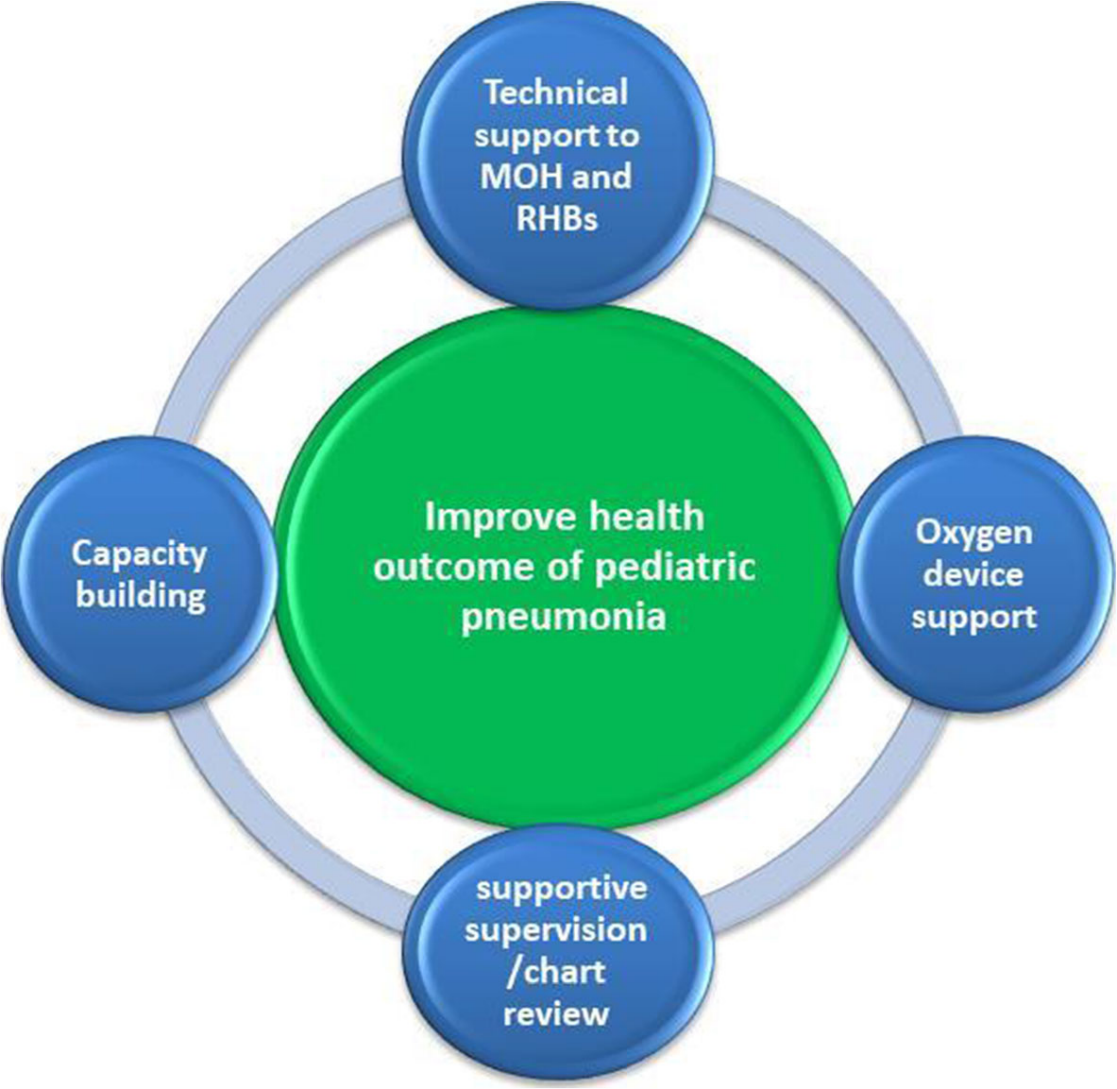
Intervention frameworks (the interventions are contributing towards improving health outcomes of pediatric pneumonia)

Between December 2015 and December 2019, a multifaceted program was implemented to improve oxygen services in Ethiopia. The program included development and formation of new policies and guidelines, procurement of oxygen equipment and pulse oximetry, capacity building of healthcare workers, and routine monitoring of program progress. A framework that illustrates the array of program interventions are presented in (Fig. 1).

### Policy and guidelines

The program provided technical support to the Ministry of Health (MOH) and Regional Health Bureaus (RHBs) to formulate a National Oxygen and Pulse Oximetry Scale-up Road Map and advocated for resources for its implementation. The road map was launched in September 2016 and served to coordinate government departments and development partners around key strategic activities that would need to be implemented in order to increase oxygen access. A national taskforce was established at the federal level to oversee the implementation of the road map.

The program also worked with the MOH and RHBs to develop technical specification guidelines and training materials for biomedical engineers, technicians, and clinical staff. Prior to the program, technical specifications for oxygen biomedical equipment did not exist; this contributed to procurement and donation of oxygen equipment unfit for Ethiopia’s context. Technical specifications for oxygen concentrators, pulse oximeters, oxygen analyzers, and pressure swing adsorption (PSA) plants were developed to guide all future procurement of these devices. Training materials specific to oxygen equipment did not exist prior to the program. Manuals on the maintenance, repair, and use of oxygen equipment for biomedical engineers and technicians, were developed as a part of implementation. Similarly, training materials on the use of oxygen equipment and pulse oximetry were also developed for clinical staff.

### Capacity building

Once training materials were developed, trainings were provided in partnership with the MOH and RHBs to 2388 healthcare workers and 326 biomedical engineers and technicians.

Additionally, focused supportive supervisions and technical support were conducted biannually in 32 hospitals that were selected to be model sites. The model sites were intentionally drawn from each region of Ethiopia. The supportive supervision teams were composed of staff from the RHBs, Ethiopian Pharmaceutical Supply Agency (EPSA), and the Clinton Health Access Initiative (CHAI). Members of the supervision team had a minimum of a first-degree qualification in health and related disciplines, with experience working at various tiers of the health system, and were experienced in supervision techniques. During the supervision visits, the supervision team observed healthcare workers diagnose hypoxemia and administer. The supervisory team provided technical support to strengthen clinical skills based on their findings, and worked with the facility staffs and management to bring improvements in the identified gaps. At the end of each visit, the team also developed action points for follow-up and implementation.

### Oxygen equipment procurement

To enhance the availability and quality of service provision, oxygen devices, including 3000 oxygen concentrators, 10 oxygen analyzers, and 5500 pulse oximeters were procured and provided to hospitals in the country. Moreover, in order to boost the national oxygen production capacity, five PSA plants were installed during the project.

### Monitoring and evaluation

To evaluate whether oxygen availability and use improved during the program period, the program team reviewed medical charts at model facilities every 6 months. All medical charts of children under-five were screened and charts with a diagnosis of pneumonia were pulled. From the medical charts of pneumonia cases, 10 charts were randomly selected from each hospital. A structured data extraction form was used to collect key variables from the medical charts, including whether there was a blood oxygen saturation (SpO2) recorded, the SpO2 level, whether oxygen was administered and routes of administration, and outcome of the child. Based on the findings of the chart reviews, onsite support and feedback were provided to the healthcare workers and management team of the facilities.

### Study design, setting, period and population

The study employed a time series design. After assessing and setting baseline values, arrays of interventions were provided and followed prospectively to assess at regular intervals the availability and functionality of pulse oximetry and oxygen devices since the end of 2015. Moreover, retrospective reviews of medical records of children between 0 and 59 months were conducted between 2017 and 2019 for hypoxemia diagnosis and oxygen therapy in the 32 model hospitals described above.

### Sampling and sample size

As noted above, 32 public hospitals were purposefully selected as model sites. The pulse oximetry and oxygen device functionality assessment has been in place every 6 months since 2016, after baseline was set in 2015. The medical records review process, for hypoxemia diagnosis and oxygen therapy assessments, began in 2017, once improvements were made in device functionality. Within each hospital, all medical charts of children under-five admitted to the paediatric ward were screened. From these medical charts, 10 were randomly selected. and a total of 320 charts reviewed in each round with total of 1600 medical charts reviewed in five rounds throughout the project. The pediatric ward admission register was utilized to create the primary list; charts were randomly selected from Medical Record Numbers (MRNs) of severe pneumonia under-five cases seen in the last six complete months. In order to randomly generate 10 medical records, the primary list (all severe pneumonia under-five cases) was divided by 10 to get K. Then, the medical records were selected from the primary list every K^th^ interval. Charts were reviewed for procedures and applications of oxygen therapy, such as oxygen check-ups, saturation records, oxygen prescription if hypoxemic, routes of administration, follow-up status, and outcomes of the child.

In addition to the medical record reviews, data were collected on the availability and functionality of oxygen devices, as well as on the knowledge and skill of healthcare workers in the same facilities.

### Data collection and management

Structured data extraction forms were developed to collect the variables of interest. The data collection was programmed into SurveyCTO (Dobility Inc., Cambridge, MA USA), an electronic data collection software, and the software application was downloaded onto tablet computers. The supervision team received a two-day training on the tool. To ensure the adequacy of mentorship and collecting quality data, supervision teams had a minimum of first-degree qualification in health and related disciplines, with significant experience working in each tier of the health system. An additional structured questionnaire file shows this in more detail [see Additional file 1].

### Data analysis

Data were cleaned using MS-Excel Office 2016. Further cleaning and preparation were also made using Statistical Packages for Social Sciences (SPSS). New variables on the availability of oxygen devices (either oxygen cylinder or concentrator) were created by either/or functions.

The study evaluated four primary outcomes: (1) the proportion of children under-five admitted with pneumonia who had a SpO2 measurement; (2) the proportion of children under-five admitted with pneumonia and with SpO2 < 90% that received oxygen; (3) the proportion of facilities with a pulse oximetry; and (4) the proportion of facilities with patients with functional oxygen. A descriptive analysis was performed to generate frequencies, tables, and charts to present key findings. Chi-squared tests were used to assess for statistical differences between consecutive follow-up periods. A *p*-value of less than 0.05 was used to determine if a relationship existed.

## Results

Of the hospitals included in the study, 15 (46.9%) were general hospitals, 10 (31.2%) were referral hospitals and 7 (21.9%) were primary hospitals. According to the health system tier of Ethiopia, these public hospitals serve an estimated catchment population of 51 million.

To assess the application of oxygen therapy, 10 under-five medical records were reviewed retrospectively every 6 months in each hospital. Of the 320 charts reviewed in end line, the percentage of children diagnosed with severe pneumonia and admitted to pediatric inpatient departments (PIPD) varied by significantly by hospital type, with 47% in general hospitals, 31% in referral hospitals, and 22% in primary hospitals (Table 1).

**Table 1:** Medical records by region and types of hospitals

Region	Number of hospitals	Medical records by type of hospital			
		Primary	General	Referral	Total
Amhara	6	20	20	20	60
Oromia	6	20	20	20	60
SNNPR	6	10	30	20	60
Tigray	6	20	20	20	60
Other regions	8	0	60	20	80
Total	**32**	**70 (22%)**	**150 (47%)**	**100 (31%)**	**320 (100%)**

**Fig. 2:**
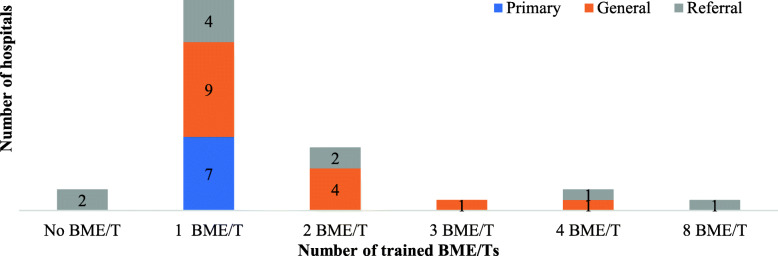
Number of trained BME/Ts on concentrator and POx maintenance by types of hospitals

As part of the initiative to increase access and availability of oxygen, onsite and offsite trainings were also provided to Biomedical Engineers/Technicians (BME/Ts), and 30 (94%) of the hospitals have trained BME/Ts. Looking into disaggregated data, primary hospitals have a maximum of one trained BME/Ts whereas, at general hospitals, there were up to nine trained BME/Ts. Overall, the highest number of trained BME/Ts were found in general hospitals followed by referral hospitals (Fig. 2).

### Availability of functional oxygen cylinder or concentrator in different departments

In general, functional availability of oxygen has shown a statistically significant increase from 62% at baseline (December 2015) [12] to 100% at end-line (December 2019) in Pediatric IPD of general and referral hospitals (*p*-value < 0.001). As presented in Table 2 and by end of the project, availability of functional oxygen was 100% in Newborn Intensive Care Units (NICU), across all hospital types. Similarly, the availability of functional oxygen was 100% at PIPDs of general and referral hospitals on the day of the visit.

**Table 2:** Availability of functional oxygen by department and types of hospitals

Department	Responses	Types of hospital					
		Primary		General +Referral		Total	
		Freq.	%	Freq.	%	Freq.	%
Pediatric IPD	Yes	7	100%	25	100%	32	100%
	No	0	0%	0	0%	0	0%
	**Total**	**7**	**100%**	**25**	**100%**	**32**	**100%**
Pediatrics Emergency	Yes	1	100%	17	89%	18	90%
	No	0	0%	2	11%	2	10%
	**Total**	**1**	**100%**	**19**	**100%**	**20**	**100%**
NICU	Yes	7	100%	25	100%	32	100%
	**Total**	**7**	**100%**	**25**	**100%**	**32**	**100%**

**Fig. 3:**
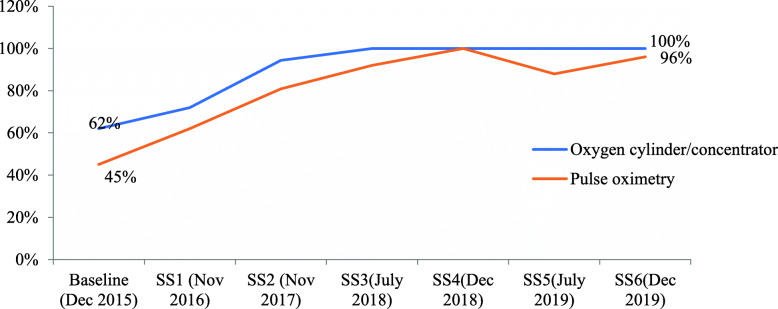
Trends in the availability of functional oxygen and POx at PIPD in general and referral hospitals

As shown in Fig. 3, the availability of functional oxygen revealed an increasing trend since baseline and has remained close to 100% since the third round Supportive Supervision (SS).

As shown in Table 3, the availability of functional pulse oximetry was 96% at PIPD of general and referral hospitals on the day of the visit. Overall, the functional availability of pulse oximetry has shown a statistically significant increase from 45% at baseline (December 2015) [12] to 96% (December 2019) in pediatric IPD of general and referral hospitals (*p-value* < 0.001).

**Table 3:** Availability of functional pulse oximetry by department and types of hospital

Department	Responses	Types of hospital					
		Primary		General +Referral		Total	
		Freq.	%	Freq.	%	Freq.	%
Pediatric IPD	Yes	6	86%	24	96%	30	94%
	No	1	14%	1	4%	2	6%
	**Total**	**7**	**100%**	**25**	**100%**	**32**	**100%**
Pediatrics Emergency	Yes	1	100%	18	94.7%	19	95%
	No	0	0%	1	5.3%	1	5%
	**Total**	**1**	**100%**	**19**	**100%**	**20**	**100%**
NICU	Yes	6	86%	24	100%	30	97%
	No	1	14%	0	0%	1	3%
	**Total**	**7**	**100%**	**24**	**100%**	**31**	**100%**

### Clinical practices of oxygen therapy

**Fig. 4:**
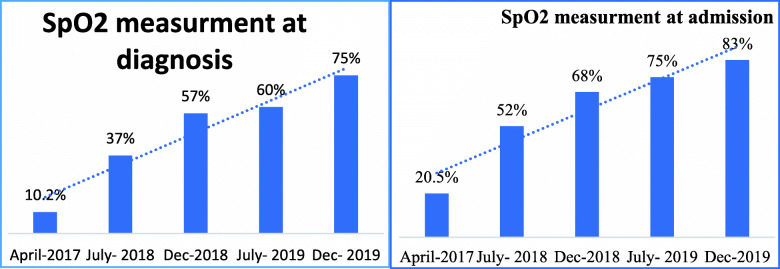
Status of POx assessment at diagnosis and admission

Of 320 medical record reviews, 241 (75%) of the children had oxygen saturation measurements using pulse oximetry at diagnosis. In addition, 266 (83%) of the children had oxygen saturation measurements using pulse oximetry after admission (in-patient units). As displayed in the graph below, SpO2 measurement at diagnosis has sharply increased from 10.2% (April 2017) to 75% (December 2019). Similarly, SpO2 measurement at admission also showed a progressive increase from 20.5 to 83%. The increments were statistically significant, both at diagnosis and admission (*p*-value < 0.001), Fig. 4.

Out of the medical records of 272 children assessed with pulse oximetry at diagnosis or any point during admission, the majority, 210 (77%), were diagnosed with hypoxemia (SpO2 < 90). Of the children with hypoxemia (210), the majority, 191 (91%), were prescribed oxygen, this includes at any time at the facility, during diagnosis, or during their stay in PIPD/pediatric emergency. The result was also statistically significant (*p-value* < 0.001).

Although there are a sizeable number of hospitals in which the prescriptions did not state flow rate, there were progressive improvements from the preceding supportive supervisions with a statistically significant increase in recording flow rates, from 13 to 53% *(p-value < 0.001).*

**Fig. 5:**
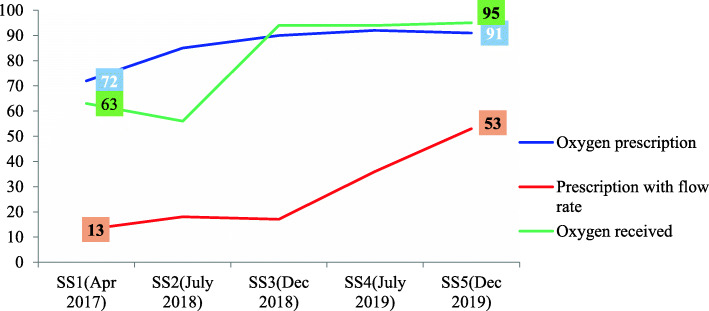
Trends in oxygen prescription, prescription with flow rate and oxygen received

In terms of receiving oxygen, of those children having hypoxemia (SpO2 < 90) at diagnosis or any point during admission, the review showed that the majority (95%) received oxygen. This is compared to only 63% of children receiving oxygen at the start of the project. This shows that there is improvement in managing children with hypoxemia. The result was also statistically significant with (*p-value* < 0.001), Fig. 5.

## Discussions

Functional availability of oxygen has shown a statistically significant increase in PIPDs of general and referral hospitals. Similarly, the functional availability of pulse oximetry has shown a statistically significant increase. Moreover, clinical practice, such as SpO2 measurement using pulse oximetry at diagnosis and admission, has also improved.

Oxygen, as an essential drug, should always be available either with a cylinder, piping, or concentrator at all critical points of care. It is a medical therapy that saves the lives of many for more than a century [13]. A study that assesses health workers perception on the barriers of oxygen therapy for pediatric patients documented that malfunctioning oxygen cylinders and concentrators, limited or no access to pulse oximetry, and lack of continued professional training were key barriers to the delivery of oxygen therapy [14]. To ensure the availability of medical oxygen, technical and capacity buildings trainings were provided to health workers and biomedical engineers/technicians in selected public hospitals. This initiative yielded promising results by contributing to the country’s effort in the reduction of deaths due to pneumonia by increasing access and availability of oxygen. This is witnessed by the functional availability of oxygen, which has shown a statistically significant increase from 62% at baseline (December 2015) [12] to 100% in December 2019 at the PIPD of general and referral hospitals *(p-value < 0.001).* Moreover, beyond enhancing the availability of medical oxygen, the overall medical oxygen practice has improved. Prescription rates increased from 72 to 91%, inclusion of flow rate on prescriptions increased from 13 to 53%, and oxygen provision increased from 63 to 95%), showing that clinical practices have improved as a result of the project interventions.

Pulse oximetry is a non-invasive device that is used to measure the oxygen saturation level in the blood as a diagnosis of hypoxemia [15]. As a standard clinical practice, pulse oximetry should be applied to all admitted children at least once during admission and for all children with pneumonia. A study reported that the availability of oximetry has increased the referral rate for severely hypoxemic children with statistically significant result [16]. Cognizant of this importance, training and regular technical supports were provided to increase access and availability of pulse oximetry in selected public hospitals. As a result, the functional availability of pulse oximetry increased from 45% at baseline (December 2015) [12] to 96% in December 2019 at PIPDs of general and referral hospitals. The increase was also statistically significant (*p*-value < 0.001).

Various studies suggest that regular technical support and supportive supervision are one of the key tasks in the management of the health system and pertinent to improve the delivery of quality services [17]. More importantly, in low-resource settings, supportive supervisions are pivotal in enhancing the motivation and performance of health workers in delivering quality services [18, 19]. Another study verified that the introduction of supportive supervision as part of capacity building and service improvement initiatives in countries like Bangladesh, Brazil, Honduras, Kenya, Nepal, and Tanzania, has registered promising results in both service quality and providers’ performance [17].

Our implementation strategies and the results achieved were in line with this research. Various researchers suggested that capacity building with regular site support can increase both the performance and motivation of health workers which in turn, ultimately improves the accessibility and quality of service delivery [17, 20, 21]. A systematic review also corroborated that the association between clinical supervision of health workers and the effectiveness of care [21]. The review reported that substantial improvement in the process and compliance of care associated with enhanced patient health outcomes [21]. This is also in agreement with our results, which demonstrated the improvement in the process of oxygen therapy provision (such as oxygen prescription, flow rate and oxygen receiving status) for pediatric patients.

Capacity building and regular supportive supervisions were conducted throughout the project life and this enhanced the availability and functionality of medical oxygen and pulse oximetry in selected public hospitals. This is substantiated by a recent study that assessed the frequency of supportive supervision and its dose-response relationship. The study found that an optimum number of regular supportive supervision has an incremental effect on service delivery improvements [22].

Furthermore, research findings also reported that the technical support and supervision of government biomedical departments strongly facilitated equipment maintenance, which substantially augments the availability of medical oxygen, which in turn enhances quality service provision [13, 23]. In line with this, the project in collaboration with national and regional health bureaus trained and provided regular technical support to biomedical engineers/technicians which contribute to the availability of medical oxygen and increase service quality in health facilities.

### Limitation of the study

Although the selected hospitals were across all the tiers of the health system and represent all regions and city administrations, the hospitals were not selected randomly. Additionally, the sample size did not also follow rigorous sampling and sample size determination procedures. However, the facilities selected were not unique – and almost all hospitals across each tier and regions have similar settings. Therefore, our findings can be extrapolated for the rest of public hospitals in Ethiopia and to other contexts with similar systems.

## Conclusions

Based on the intervention results, we can conclude that multifaceted approaches targeting health care workers’ capacity, increased device procurement, and on-site device maintenance with on-site mentorship, can improve the availability of medical oxygen and pulse oximetry and clinical practice of oxygen therapy in health facilities. Therefore, ensuring device availability along with regular technical support and close follow-up of healthcare workers and facilities are critical; these actions must be expanded to bring large scale improvement and health systems transformation.

## Supplementary Information

**Additional file 1.** Medical Oxygen and Pulse Oximetry Supportive Supervision Tool.

## Data Availability

The datasets used and/or analyzed during the current study are available from the corresponding author on reasonable request.

## Abbreviations

BME/Ts: Biomedical Engineers/Technicians
EPHI: Ethiopian Public Health Institute
EPSA: Ethiopian Pharmaceutical Supply Agency
HCWs: Health Care Workers
MoH: Ministry of Health
NICU: Newborn Intensive Care Unit
PIPD: Pediatric Inpatient Departments
POx: Pulse Oximetry
RHBs: Regional Health Bureaus
