# Supplementary material for: Changes in the availability of medical oxygen and its clinical practice in Ethiopia during a national scale-up program: a time series design from thirty-two public hospitals
Source: BMC Pediatr. 2021 Oct 14;21:451. doi: 10.1186/s12887-021-02844-4 (PMC8515671; doi:10.1186/s12887-021-02844-4)
Supplement: Supplementary file 1 — Additional file 1. Medical Oxygen and Pulse Oximetry Supportive Supervision Tool. [file 12887_2021_2844_MOESM1_ESM.docx]

**Medical Oxygen and Pulse Oximetry Supportive Supervision Tool**

| **Instructions: Please begin by introducing yourself and explaining the basic details of the visit** |
| --- |

**SECTION I: GENERAL INFORMATION**

| **NO.** | **QUESTION** | **RESPONSE CODE** | **SKIP** |
| --- | --- | --- | --- |
|  | Date of visit  (use Gorgonian calendar) | _______/____/______  *DD/MM/YYYY* |  |
|  | Full Name of supervisors | A.  B.  C.  D. |  |
|  | INSTITUTION OF supervisors | A.  B.  C.  D. |  |
|  | Telephone number of supervisors  *The telephone number should have 9 digits. Do not enter 251 or 0.* | A.  B.  C.  D. |  |
|  | Name of the Hospital | _______________________________________ |  |
|  | Type of the hospital | Primary 1  General 2  Referral/specialized/University 3 |  |
|  | Region | _______________________________________ |  |
|  | Zone | _______________________________________ |  |
|  | Town | _______________________________________ |  |
|  | Telephone number of THE HOSPITAL  *Record the telephone number that is owned by the Hospital. The telephone number should have 9 digits. Do not enter 251 or 0.* | _______________________________________ |  |
|  | Contact information of THE CHIEF Executive Officer(CEO)  [A] Name  [B] Telephone number  The telephone number should have 9 digits. Do not enter 251 or 0.  [C] Email (Optional) | A.  B.  C. |  |
|  | Contact information of the Medical director or Chief Clinical Officer (CCO)  [A] Name  [B] Telephone number  The telephone number should have 9 digits. Do not enter 251 or 0.  [C] Email (Optional) | A.  B.  C. |  |
|  | Contact information of the Biomedical Engineer/ Technician (Head of the unit)  [A] Name  [B] Telephone number  The telephone number should have 9 digits. Do not enter 251 or 0.  [C] Email (Optional) | A.  B.  C. |  |
|  | Distance of THE HOSPITAL from:  [A] Regional city  [B] Zone town  [ C] the nearest referral hospital (FOR primary & General hospitals)  *If don’t know, please state your expert estimation*  *If in the same town/city, write 0 (zero) km* | A. Regional city ____________________KM  B. Zone town ______________________KM  C. Nearest Referral hospital ___________KM |  |

**SECTION II: MEDICAL OXYGEN SERVICE AVAILABILITY AND USE**

| **NO.** | **QUESTION** | **RESPONSE CODE** | **SKIP** |
| --- | --- | --- | --- |
|  | what sources of oxygen are used at this hospital?  *Mark all responses mentioned.* | Oxygen cylinders A  Oxygen concentrators B  Piped oxygen from a plant C | A🡪O2  B🡪O19  C🡪O37 |
|  | Is there functional cylinder available in the following units?  *NA will be selected if the department or the unit is not available in the facility.* | Yes No NA  [A] Pediatric OPD 1 2 3    [B] Pediatric IPD 1 2 3  [C] Pediatrics Emergency 1 2 3  [D] Emergency 1 2 3    [E] NICU 1 2 3  [F] ICU (all types) 1 2 3  [G] Maternity ward and L&D 1 2 3  [H] Gyn Ward 1 2 3  [I] OR and recovery 1 2 3  [J] Surgical Ward 1 2 3  [K] Medical ward 1 2­­­­­­­ ­­ 3  [L ] Other (specify) 1 2 3 |  |
|  | if there is no cylinder in any of the units at O3, what are the reasons?  *Ask and circle all that apply:* | Yes No  [A] Shortage of budget 1 2  [B] Sharing with other Units 1 2  [C] Sent for refilling/not refilled 1 2  [D] Source of O_2_ in the unit is concentrator 1 2  [D] Others, Specify below 1 2  ______________________________________ |  |
|  | if there is non-functional cylinder in any of the units at O3, what are the reasons?  *Ask and circle all that apply:* | Yes No  [A] Not reported to BME/T 1 2  [B] Lack of spare part 1 2  [C] Lack of BME/T 1 2  [D] BME’s maintenance skill gap 1 2  [E] Not repairable (Obsolete) 1 2  [F] Others, Specify below 1 2  ______________________________________ |  |
|  | Where do you refill your cylinders usually?  *Write the name of the refilling center* | ______________________________________ ______________________________________ |  |
|  | do you have a written agreement with the refilling centre?  *Verify through observation* | Yes 1  No 2 |  |
|  | what is the distance from your hospital to the nearest refiling centre? | Distance: _________________________K.M |  |
|  | does the hospital have cylinder refilling schedule? | Yes 1  No 2 |  |
|  | how many days does it usually take between sending the oxygen cylinders to be refilled and receiving them back? | Number of Days _________________________ |  |
|  | On average, how many cylinders are delivered to your facility each month? | Cylinders delivered per month______________ |  |
|  | average monthly oxygen consumption in volume? | Average volume of oxygen ______________M^3^ |  |
|  | Are all oxygen cylinders coded according to Ethiopian standard?  *Verify that all cylinders are painted with white color* | Yes 1  No 2 |  |
|  | On average, what is the price you pay to re-fill one oxygen cylinder (excluding transportation)? | Price per cylinder__________________ ETB  OR  Price per M^3^______________________ETB |  |
|  | is there mechanism of checking concentration (quality) of oxygen during accepting the cylinders using oxygen analysers? | Yes 1  No 2 |  |
|  | are there any standard oxygen consumption recording formats at the hospital?  *Verify through observation of the standard consumption format per facility.* | Yes, verified 1  No 2 |  |
|  | does the hospital record oxygen consumption on recording formats?  *Verify through observation.* | Yes, verified 1  No 2 |  |
|  | Is there anyone responsible focal person assigned for oxygen logistics management in this facility? | Yes, fulltime 1  Yes, but not fulltime 2  No, 3 |  |
|  | what are the main challenges for the re-filling process?  *Ask and circle all that apply:* | Yes No  [A] Cost of refiling cylinders 1 2  [B] Distance to refiling center 1 2  [C] Transportation mechanism 1 2  [D] Others, Specify below 1 2  ______________________________________ |  |
|  | Is/are there functional concentrator/s available in the following units?  *NA will be selected if concentrators are not selected as source of oxygen at O1.* | Yes No NA  [A] Pediatric OPD 1 2 3    [B] Pediatric IPD 1 2 3  [C] Pediatrics Emergency 1 2 3  [D] Emergency 1 2 3    [E] NICU 1 2 3  [F] ICU (all types) 1 2 3  [G] Maternity ward and L&D 1 2 3  [H] Gyn Ward 1 2 3  [I] OR and recovery 1 2 3  [J] Surgical Ward 1 2 3  [K] Medical ward 1 2­­­­­­­ ­­ 3  [L ] Other (specify) 1 2 3 |  |
|  | if there is no oxygen concentrator in any of the units at O19, what are the reasons?  *Ask and circle all that apply:* | Yes No  [A] Shortage of budget 1 2  [B] Sharing with other Units 1 2  [C] Others, Specify below 1 2  ______________________________________ |  |
|  | if there is non-functional oxygen concentrator in any of the units at O19, what are the reasons?  *Ask and circle all that apply:* | Yes No  [A] Not reported to BME/T 1 2  [B] Lack of spare part 1 2  [C] Lack of BME/T 1 2  [D] BME’s maintenance skill gap 1 2  [E] Not reparable (Obsolete) 1 2  [F] Others, Specify below 1 2  ______________________________________ |  |
|  | is there a back-up power source the concentrator can use (e.g. generator) in case the hospital loses electricity? | Yes 1  No 2 |  |
|  | is there mechanism of checking concentration or quality of oxygen by using oxygen analyser?  *(Especially during maintenance )* | Yes 1  No 2 |  |
|  | Is there functional pulse oximetry available in the following units?  *NA will be selected if pulse oximetry service is not available at all in the facility.* | Yes No NA  [A] Pediatric OPD 1 2 3    [B] Pediatric IPD 1 2 3  [C] Pediatrics Emergency 1 2 3  [D] Emergency 1 2 3    [E] NICU 1 2 3  [F] ICU (all types) 1 2 3  [G] Maternity ward and L&D 1 2 3  [H] Gyn Ward 1 2 3  [I] OR and recovery 1 2 3  [J] Surgical Ward 1 2 3  [K] Medical ward 1 2­­­­­­­ ­­ 3  [L ] Other (specify) 1 2 3 |  |
|  | if there is no pulse oximeter in any of the units at O24, what are the reasons?  *Ask and circle all that apply:* | Yes No  [A] Shortage of budget 1 2  [B] Sharing with other Units 1 2  [C] Others, Specify below 1 2  ______________________________________ |  |
|  | if there is non-functional pulse oximeter in any of the units at O24, what are the reasons?  [A] Not reported to BME/T  [B] Lack of spare part  [C] Lack of BME/T  [D] BME/T’s maintenance skill gap  [E] Not reparable (Obsolete)  [F] Others, Specify below  *Ask and circle all that apply:* | Yes No  [A] Not reported to BME/T 1 2  [B] Lack of spare part 1 2  [C] Lack of BME/T 1 2  [D] BME’s maintenance skill gap 1 2  [E] Not reparable (Obsolete) 1 2  [F] Others, Specify below 1 2  ______________________________________ |  |
|  | Is there any biomedical engineer or technician in the facility? | Yes 1  No 2 | 1🡪O28  2🡪O34 |
|  | Total number of biomedical technicians or biomedical engineer in currently working in the hospital? | A. Number of biomedical engineers___________  B. Number of biomedical technicians___________ |  |
|  | if available, how many of them are trained on oxygen concentrator and pulse oximetry maintenance?   1. biomedical engineers 2. biomedical technicians | A. # of trained biomedical engineers___________  B. # of trained biomedical technicians___________ |  |
|  | Are there oxygen device maintenance job aids or SOPs for technicians and BMEs? | Yes 1  No 2 |  |
|  | does the hospital have preventive maintenance schedule? | Yes 1  No 2 |  |
|  | does the hospital have notification and work order system in place for medical equipment maintenance?  *Verify through observation.* | Yes, verified 1  No 2 |  |
|  | does the hospital have maintenance kit for oxygen device? | Yes, verified 1  No 2 |  |
|  | Are the following spare parts of pulse oximetry adequately available in your stock? | Yes No  [A] Batteries for POx 1 2  [B] Replacement probes 1 2  [C] Others, Specify below 1 2  ______________________________________ |  |
|  | Are the following spare parts or replacement parts of concentrator adequately available in your stock? | Yes No  [A] Filters 1 2  [B] Fuse 1 2  [C] Battery (9 V) 1 2  [D] Compressor 1 2  [E] Sieve beds 1 2  [F] Valve spares 1 2  [G] Capacitor 1 2  [H] Others, specify 1 2  ________________________________________ |  |
|  | Are the following spare parts or replacement parts of cylinder adequately available in your stock? | Yes No  [A] Gauge 1 2  [B] Regulator 1 2  [C] Flow meter 1 2  [D] Humidifier 1 2  [E] Others, specify 1 2  ________________________________________ |  |
|  | IS there a physician/ nurse in the hospital trained on medical oxygen therapy | Yes 1  No 2 |  |
|  | Under which situations are pulse oximeters used in this facility?  *circle all that apply* | Pre O2 administering assessment A  During O2 administration (monitoring) B  To make decision for stopping oxygen C  Other (specify) X  Don’t know Z |  |
|  | are there any job aids or sops for providing oxygen therapy at the hospital?  *If yes, verify through observation.* | Yes, verified 1  No 2 |  |
|  | Does the facility train all clinical staff who use oxygen equipment in basic concentrator maintenance or troubleshooting? | Yes, all staff who use the equipment are trained 1  Yes, but only some of the staff who use the equipment are trained 2  No, clinical staff are not trained 3 |  |
|  | Are oxygen related adverse events (overdose, toxicity, Retinopathy of prematurity) observed at this hospital? | Yes 1  No 2 |  |
|  | If yes, how many oxygen related adverse events (ADEs) were reported at the hospital within the last 12 months? | # of reported O2 related ADEs _______ |  |

G1. Date of visit------ G2. Region ---------G3. Name of Hospital_________________ G4. Type of Hospital_______________ G5. Card #

Review 10 randomly selected medical records of under 5 patients with severe pneumonia seen in the past 6 months **(juNE-OCTOBER, 2019)**:

|  | Criteria | Write 1 if Yes, write 2 if No or write 3 if not applicable (the stated service is not available) | | | | | | | | | |
| --- | --- | --- | --- | --- | --- | --- | --- | --- | --- | --- | --- |
|  |  | **MR-1** | **MR-2** | **MR-3** | **MR-4** | **MR-5** | **MR-6** | **MR-7** | **MR-8** | **MR-9** | **MR-10** |
| 1 | Did the child get a POx assessment at triage? |  |  |  |  |  |  |  |  |  |  |
| 2 | Did the child get a POx assessment at diagnosis? |  |  |  |  |  |  |  |  |  |  |
| 3 | If yes to Q2, did the child have hypoxemia (SPO2 < 90) at diagnosis? |  |  |  |  |  |  |  |  |  |  |
| 4 | If yes to Q3, Was the child prescribed oxygen as part of the initial clinician’s  order at diagnosis? |  |  |  |  |  |  |  |  |  |  |
| 5 | Did the child get a POx assessment at any point during admission? |  |  |  |  |  |  |  |  |  |  |
| 6 | If yes to Q5, did the child have hypoxemia (SPO2 < 90) at any point during admission? |  |  |  |  |  |  |  |  |  |  |
| 7 | Was the child prescribed oxygen at any time (including at diagnosis)  during the stay (emergency and/or IPD)? |  |  |  |  |  |  |  |  |  |  |
| 8 | If yes to Q7, does the prescription state the mode of delivery of oxygen?  (Nasal prong, Catheter or Facemask) |  |  |  |  |  |  |  |  |  |  |
| 9 | If yes to Q7, does the prescription state flow rate? |  |  |  |  |  |  |  |  |  |  |
| 10 | If yes to Q7, does the prescription mention target SPO2? |  |  |  |  |  |  |  |  |  |  |
| 11 | If yes to Q7, did the prescriber mention frequency of monitoring SPO2? |  |  |  |  |  |  |  |  |  |  |
| 12 | Was the child actually received oxygen? |  |  |  |  |  |  |  |  |  |  |
| 13 | Was the child SPO2 monitored at least once per day? |  |  |  |  |  |  |  |  |  |  |
| 14 | Was the oxygen therapy stopped after at least two records of SPO2 > 90%? |  |  |  |  |  |  |  |  |  |  |
| 15 | Status at the end –Improved? Referred? Left against medical advice? Died?  Write **A** for Improved, B for Referred, **C** for Left against medical advice, **D** for Died |  |  |  |  |  |  |  |  |  |  |

| **Instructions: Thank the hospital staff for their time. Provide any final feedback to them regarding the supportive supervision visit.** |
| --- |

**SECTION III: END**

| **NO.** | **QUESTION** | **RESPONSE CODE** | **SKIP** |
| --- | --- | --- | --- |
| COMMENT | Thank you for your time. This concludes our visit. Are there any other comments you would like to tell us?  *Write on your notebook for narrative report, but this will not be written on tablets.* |  | |
| GPS | GPS location of the Facility  *Make sure the device's GPS is turned on and you are standing outdoors when recording the GPS location and capture the data when the accuracy is possibility below 10.* |  | |
